# Functional characterization and pharmacochaperone rescue of a novel SLC6A1 variant associated with developmental epileptic encephalopathies

**DOI:** 10.1038/s41598-026-72601-z

**Published:** 2026-10-07

**Authors:** Ziang Debbie Song, Melissa B. DeLeeuw, Despoina Mandelenaki, Wangzhen Shen, Ria S. Eda, Juexin Wang, Jing-Qiong Kang

**Affiliations:** 1 Department of Neurology, Vanderbilt University Medical Center, Nashville, TN 37232 USA; 2 Children’s Neuroscience, Evelina London Children’s Hospital at Guy’s and St Thomas’ NHS Foundation Trust, London, UK; 3 The Neuroscience Program, Vanderbilt University Brain Institute, Nashville, USA; 4 Department of Biomedical Engineering and Informatics, Luddy School of Informatics, Computing, and Engineering, Indiana University Indianapolis, Indianapolis, IN 46202 USA; 5 Department of Pharmacology, Vanderbilt University, Nashville, USA; 6 Vanderbilt Kennedy Center of Human Development, Vanderbilt University, Nashville, TN 37232 USA; 7 Department of Neurology & Pharmacology, Vanderbilt Brain Institute, Vanderbilt University Medical Center, Vanderbilt Kennedy Center of Human Development, 6147 MRBIII, 465 21st Ave. South, Nashville, TN 37232 USA

**Keywords:** Artificial intelligence (AI), Epilepsy, GABA transporter 1 (GAT-1), Protein stability, Endoplasmic reticulum (ER) retention, 4-Phenylbutyrate (PBA), Diseases, Drug discovery, Genetics, Neurology, Neuroscience

## Abstract

Mutations in *SLC6A1*, encoding the γ-aminobutyric acid (GABA) transporter 1 (GAT-1), are associated with a spectrum of neurodevelopmental disorders, including epilepsy syndromes, intellectual disability (ID), and autism. An important question is whether the impact of these mutations can be predicted at scale using artificial intelligence (AI), and how such predictions correlate with experimental findings. In this study, AI was explored as a predictive tool, not as a replacement for data collected experimentally. We reported clinical presentations including seizures and EEG features, autism related phenotypes and motor skills in a novel variant c.1069G > A (p.Ala357Thr, A357T) in two siblings. We used both AI tools and experimental approaches to characterize this novel mutation in the *SLC6A1* gene and compared it with another variant in the same amino acid, c.1070 C > T (p.Ala357Val, A357V). Both AI and experimental approaches indicate that the GAT-1(A357T) and (A357V) mutations destabilize the global protein conformation and have increased localization inside the endoplasmic reticulum (ER). Additionally, the presence of the mutant GAT-1 made the wildtype GAT-1 less mature. Radioactive ^3^H-labeled GABA uptake assay indicated the mutation drastically reduced the function of the GAT-1(A357T) compared with the wildtype across cell types and pharmacochaperones such as 4-phenylbutyrate rescued the trafficking and function of the mutant GAT-1. Common and differential clinical phenotypes were identified in the siblings carrying the same variant. Mutant GAT-1 caused increased ER retention and impaired the trafficking of wild type GAT-1. PBA treatment rescued the mutant GAT-1 protein expression and GABA uptake function. AI-assisted in silico protein structural modeling and experimental approaches consistently suggest that the A357T and A357V variants destabilize global GAT-1 protein conformation. AI-assisted protein stability predictions provide supportive structural insight but cannot replace experimental assessment of GAT-1 expression and function.

**Supplementary Information:**

The online version contains supplementary material available at https://doi.org/10.1038/s41598-026-72601-z.

## Introduction

Gamma-aminobutyric acid (GABA) transporter 1 (GAT-1) encoded by SLC6A1 is one of the primary transporters for GABA. It clears GABA from the synapse and extracellular space1, which is essential in maintaining the homeostasis of excitation and inhibition in the central nervous system. Pathogenic variants in SLC6A1 were first identified as a cause of epilepsy with myoclonic-atonic seizures, and related generalized epilepsy syndromes^1−4^, and have since been associated with a broad phenotypic spectrum including developmental and epileptic encephalopathies (DEEs), intellectual disability, autism spectrum disorder, and attention-deficit/hyperactivity disorder^5^. We^4−7^ have studied the impact of mutation on the mutant GAT-1 function and trafficking in cell models of neurons and astrocytes as well as knockin mouse models bearing representative mutations^1–3^. We identified that reduced GABA uptake functionality is common cross mutations with various phenotypes. Studies from other groups^8,9^ are consistent with our findings. Reduced GABA uptake function is due to impaired GAT-1 protein trafficking and reduced protein stability6,10. Importantly, we have identified 4-phenylbutyrate, previously known as a chemical chaperone inducer and HDAC inhibitor used for urea cycle disorders^11,12^, can restore the GABA uptake and mitigate seizures in the cell and mouse models^13^ as well as human patients bearing the SLC6A1 variants^3^. Considering the ongoing clinical trial (NCT04937062) and early promise of 4-phenylbutyrate in SLC6A1 variant mediated developmental and epileptic encephalopathies (DEEs), it is essential to systematically evaluate the pathogenicity and therapeutic responsiveness of each SLC6A1 variant to enable precision-guided use of Ravicti (glycerol phenylbutyrate) in patients.

Emerging artificial intelligence (AI) technologies, including machine learning and generative AI, offer powerful, data-driven approaches for evaluating the impact of genetic mutations on protein structure and function^14,15^. Established structure-based computational platforms, including I-TASSER, MAESTROweb, DynaMut, and aggregation-prediction tools such as PASTA, have been widely used to predict mutation-induced changes in protein conformation, flexibility, stability, and aggregation propensity. This transformative capability holds promise for identifying novel therapeutic targets and advancing precision drug discovery strategies in DEEs^16^. We have extensively studied the pathophysiology of GABA_A_ receptors and GAT-1 mutations with experimental approaches^5–8^. Amino acid substitutions in both mutant GABA_A_ receptors or GAT-1 frequently lead to structural and chemical alterations that disrupt protein conformation, resulting in a loss or modification of function. These functional disruptions in the mutant protein can trigger a cascade of cellular and network-level changes, ultimately manifesting as seizures and abnormal neurobehavioral phenotypes^7^. Although artificial intelligence-based prediction tools can assist in evaluating the potential impact of genetic variants, these approaches cannot replace experimental validation, which remains essential for defining functional consequences and therapeutic relevance in *SLC6A1*-related disorders. Thus, a combined experimental and AI-assisted approach will empower our understanding of disease pathophysiology, leading to more targeted and effective treatment development on a large scale.

In the current study, we utilized both AI-assisted and experimental approaches and characterized a novel mutation in the first pedigree with two siblings of opposite genders from the same parents. We characterized the EEG patterns and the phenotypes of DEEs in the affected brother and sister, including a thorough evaluation of patient disease history, epilepsy, motor deficit, autism and attention deficit hyperactivity disorder (ADHD) phenotypes. We evaluated the impact of the mutation (A357T) as well as the other mutation that is recurring in the same nucleotide (A357V). We evaluated the impact of the mutation with AI tools and characterized the mutant protein trafficking and function in different cell types including mouse neurons and astrocytes. We evaluated the transporter function with ^3^H radioactive GABA uptake assay and determined the protein expression and subcellular localizations via biochemical assays and confocal microscopy, in both heterologous cells and human iPSC derived cortical neuron and astrocytes overexpressing the transporters. This study provides insights into how AI-assisted structural prediction correlates the pathogenicity of the impact of the mutant protein. Additionally, we compared the common and differential clinical presentations in the affected brother and sister. Importantly, we determined the functional rescue of the mutant protein with upregulation of ER chaperones via pharmacological and genetic approaches.

## Methods

### Patient history, genetic diagnosis and clinical phenotypes

The patients and their unaffected family members were evaluated at the Evelina London children’s hospital, part of Guy’s and St. Thomas NHS Trust. The collected clinical data included age of seizure onset, a detailed developmental history, seizure types and frequency, response to antiepileptic drugs (AEDs) as well as EEG features and neuropsychiatric comorbidities. Video electroencephalography (EEG) in both patients was conducted in Department of Electroencephalopathy at Young Epilepsy, Kent, UK and was examined repeatedly, and the results were reviewed by qualified electroencephalographers. Autism spectrum disorder (ASD) and attention deficit hyperactivity disorder (ADHD) symptoms were assessed using standard assessment tools. Written informed consent for the sharing of clinical and genetic information was obtained from the parents.

### Genetic data analysis

Saliva samples of the patients and shared parents were collected and evaluated using the CLIA-approved clinical Invitae Comprehensive Epilepsy Gene Panel (Invitae, USA). The *SLC6A1* c.1069G > A (p. Ala357Thr) variant of unknown significance (VUS) was identified in both the brother and sister but not in their unaffected mother and father.

### The cDNAs for coding GABA transporter 1

The plasmid cDNA encoding enhanced yellow fluorescent protein (EYFP)-tagged GAT-1 was sub-cloned into the expression vector pCMV as in previous studies^7,13,17^. HA tagged GAT-1 was generated by inserting HA before the start of the encoding sequence of human GAT-1. Replications of patient GAT-1 mutations were cloned via a standard molecular cloning process. QuikChange Site-directed Mutagenesis kit was utilized to introduce the GAT-1(A357T) into the wildtype GAT-1 coding sequence. The product from polymerase chain reaction was transformed using DHα competent cells and finally plated. A clone was chosen and grown overnight, replicating the cDNA. The GAT-1(A357T) mutation was confirmed by DNA sequencing. Both the wildtype and the mutant cDNAs were prepared with Qiagen Maxiprep kit.

### Polyethylenimine (PEI) transfection

Standard transfection protocols were performed using human embryonic kidney 293T (HEK293T) cells purchased from American Type Culture Collection (ATCC). 24 h before transfection HEK293T cells were split equally into plates. For radiolabeling GABA uptake,1 µg of the cDNAs with PEI at a ratio of 1:2.5 µl was transfected in a 35 mm dish. The cDNAs were combined with Dulbecco modified Eagle medium (DMEM) and a PEI/DMEM mixture. For total protein expression, 3 µg cDNAs were used for transfection. In co-expression experiments, WT HA-hGAT-1 and variant YFP-hGAT-1 were co-transfected at 1:1 ratio. Transfected HEK293T cells were incubated for 48 h. After incubation, proteins were harvested as described below.

### Western blot analysis of total GAT-1 protein

Live transfected HEK293T cells were washed with phosphate buffered saline (1×PBS, pH 7.4) 3 times and then cells were lysed in RIPA buffer (20 mM Tris, 20 mM EGTA, 1 mM DTT, 1 mM benzamidine), supplemented with 0.01 mM PMSF, 0.005 µg/mL leupeptin, and 0.005 µg/mL pepstatin for 30 min at 4º C. The samples were then subject to protein concentration determination and followed by SDS-PAGE. Membranes were incubated with primary rabbit polyclonal antibodies against GAT-1 (Alomone Labs, AGT-001 or Synaptic System, 274102 at 1:200 dilution).

### Human astrocytes and neurons used for transfection were differentiated from induced pluripotent stem cells (iPSCs)

The human induced pluripotent stem cell (iPSC) line was purchased from Thermo Fisher (A18945). We followed our previous protocol used for the astrocyte and neuron differentiation from the *SLC6A1*(S295L) patient cell line^7,17^. We first prepared the neural progenitor cells (NPCs) and then differentiated the NPCs into astrocytes or neurons. The differentiation of NPCs was induced with the STEMdiff SMADi Neural induction kit from STEMCELL. The differentiation of astrocytes was initiated using the Astrocyte medium (ScienCell) for 27–30 days (doi: https://doi.org/10.1101/2020.04.21.054361), and the cells were passaged at ~ 70% confluence. The cortical inhibitory neurons were prepared following previous reports^28^. The neurons were harvested for experiments at day 60–65 after differentiation. Neuronal differentiations were initiated from the NPCs at day 10 from passage 2 after neuronal induction. Neuronal identity was validated by immunostaining with NeuN, DLX, synapsin and synaptophysin as in previous study^1^. Differentiation of human astrocytes started from NPCs at day 5 of passage 1 (P1). Astrocytes were passaged at ~ 70% confluence and were validated based on the protocol described in our previous study by staining with S100β and Glial Fibrillary Acidic Protein (GFAP)^12^. A total of 1 µg of cDNAs were transfected in one 35-mm^2^ dish for both neurons and astrocytes with lipofectamine.

### Radioactive ^3^H-labeled GABA uptake assay

The radioactive ^3^H-labeled GABA uptake assay in HEK293T, human astrocytes and neurons was modified from previous studies^7,13,17^. For all different cells, cells were cultured in 35 mm^2^ dishes but transfected and assayed accordingly based on our lab protocols. For HEK293T cells, cells were seeded at a density of 150,000 cells 3 days before the GABA uptake experiment in DMEM with 10% fetal bovine serum and 1% penicillin/streptomycin. The culture of human astrocytes and neurons were detailed above. The cells were then transfected with equal amounts of the wildtype or the mutant GAT-1 cDNAs (1 µg) with PEI at a ratio of 1 µg cDNA:2.5 µl of PEI for each condition at 24 h after cell seeding. GABA uptake assay was carried out 48 h after the transfection. The cells were incubated with preincubation solution for 15 min and then incubated with preincubation solution containing 1µci/ml ^3^H GABA and 10 µM unlabeled GABA for 30 min at room temperature. After washing, the cells were lysed with 0.25 N NaOH for 1 h. Acetic acid glacial was added and lysates were then determined on a liquid scintillator with QuantaSmart. The flux of GABA (pmol/µg/min) was averaged with at least triplets for each condition at each transfection. The average counting was taken as *n* = 1. The untransfected condition was taken as a baseline that was subtracted from both the wildtype and the mutant conditions. The pmol/µg/min in the mutant was then normalized to the wildtype from each experiment, which was arbitrarily taken as 100%. All experimental protocols involving the use of ^3^H-labeled GABA for radioactive GABA uptake assays were approved by the Vanderbilt University Medical Center (VUMC) Radiation Safety and were conducted in accordance with VUMC radiation safety guidelines.

### Live cell confocal microscopy and image acquisition

For assessment of ER retention and extracellular localization of the mutant protein, HEK293T cells expressing wildtype or mutant GAT-1 were treated with pharmacological agents or vehicle control for identical durations prior to imaging. Cells with different treatment conditions were imaged at the same post-transfection time point using identical acquisition settings to allow direct comparison of subcellular localization patterns. ER retention was evaluated by quantifying the proportion of GAT-1 fluorescence colocalizing with the ER marker, while total GAT-1 fluorescence intensity was measured to assess relative protein abundance and trafficking status across conditions.

Live cell confocal microscopy was performed using an inverted Zeiss laser scanning microscope (Model 510) with a 63 × 1.4 NA oil immersion lens, 2-2.5 × zoom, and multi-track excitation. HEK 293T cells were plated on poly-D-lysine-coated, glass-bottom imaging dishes at the density of 1–2 × 10^5^ cells and cotransfected with 0.5 µg of the wildtype or the mutant GAT-1 cDNAs and 0.5 µg pECFP-ER cDNA with PEI based on our standard lab protocol. Cells were examined with excitation at 458 nm for ECFP, 514 nm for EYFP. All images were single confocal sections averaged from 8 times to reduce noise, except when otherwise specified^18^. The images were acquired using a LSM 510 invert confocal microscope with 63X objective.

### Protein structural modeling and machine learning tools

We simulated the impact of the A357T and A357V mutations on the GAT-1 protein with multiple machine learning tools as in previous study^19^. The crystal structure of human GAT-1 (PDB ID: 7Y7W) was retrieved from the RCSB Protein Data Bank^9^. This structure was selected for its high resolution (2.9 Å) and complete coverage of the mutation site A357. Point mutations A357T and A357V were introduced in silico, and DynaMut2 was used to visualize tertiary structural changes and to predict ΔΔG and residue-level flexibility based on normal mode analysis^10^. mCSM estimated the stability impact of each mutation using graph-based signatures which encode atomic environment^11^. DUET integrated predictions from mCSM and SDM using a consensus regression model to improve predictive accuracy^12^. SDM assessed the impact of each mutation by calculating environment-specific substitution frequencies derived from homologous structures^13^.

The same structural template was used across models for consistency, and ΔΔG results were compared across tools. The effects of each mutation were interpreted in the context of polarity mismatch, steric hindrance, and hydrophobic packing.

### Data analysis

Numerical data were expressed as mean ± SEM. Proteins were quantified by Odyssey software and data were normalized to loading controls and then to wildtype GAT-1, which was arbitrarily taken as 1 in each experiment. The radioactivity of GABA uptake was measured in a liquid scintillator with QuantaSmart. The flux of GABA (pmol/µg/min) in the wildtype GAT-1 samples was arbitrarily taken as 100% each experiment. The fluorescence intensities from confocal microscopy experiments were determined using MetaMorph imaging software and the measurements were carried out in ImageJ as modified from previous description^2,14^. For statistical significance, we used one-way analysis of variance (ANOVA) with Newman-Keuls test or Student’s unpaired *t*-test. In some cases, one sample *t*-test was performed (GraphPad Prism, La Jolla, CA), and statistical significance was taken as *p* < 0.05.

## Results

### Mutation analysis identified SLC6A1(A357T) variation in two siblings

**Fig. 1:**
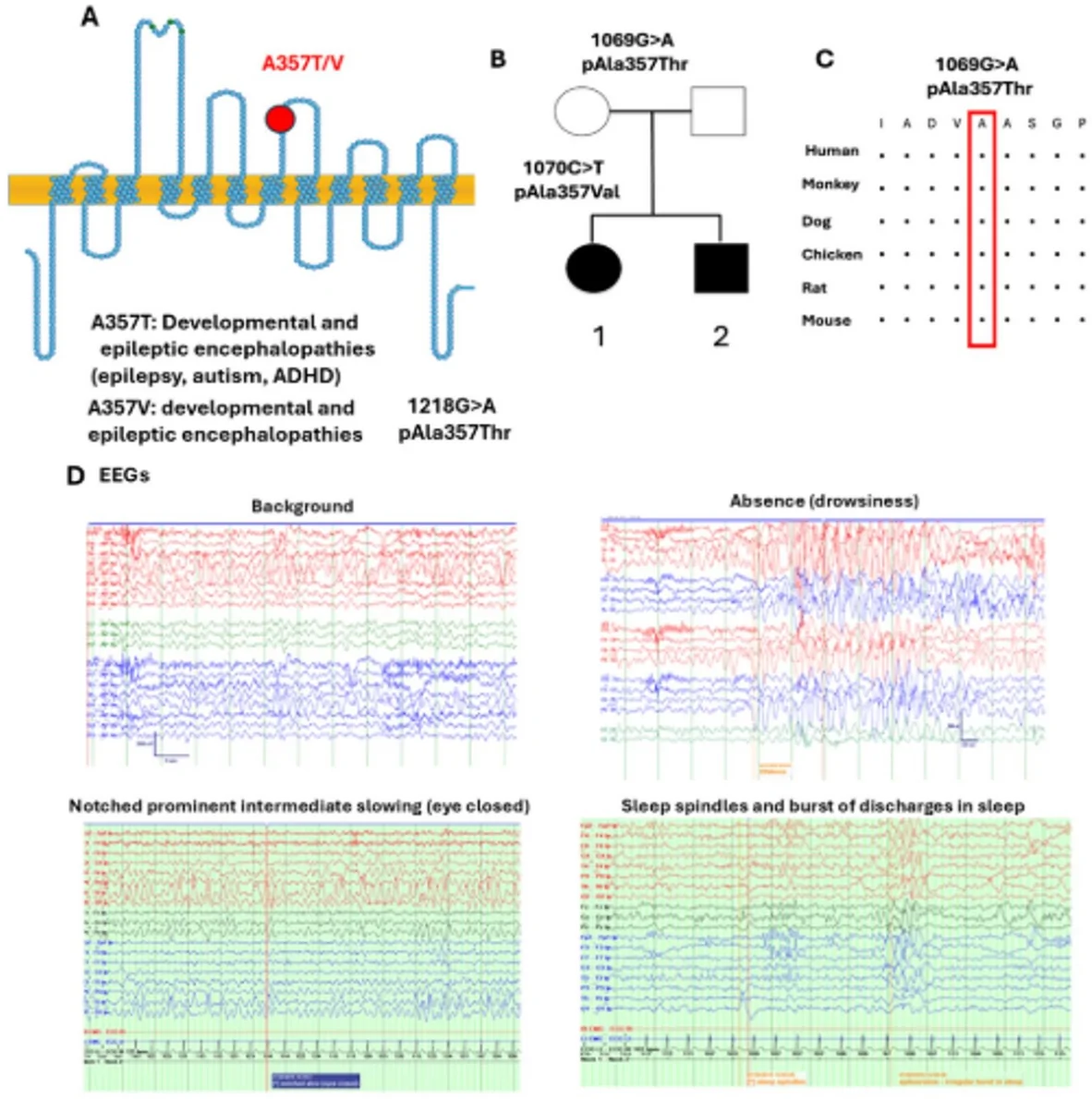
The pedigree of a novel *SLC6A1* mutation GAT1(A357T) associated with developmental and epileptic encephalopathies and the EEG patterns in affected brother and sister. **(A).** Schematic representation of GAT-1 protein topology and locations of GAT-1 variants previously identified in patients associated with developmental and epileptic encephalopathies (DEEs). It is predicted that GAT-1 contains 12 transmembrane domains. A357T is located at the extracellular domain between transmembrane 7 and 8of the GAT-1 protein (Unipro P30531 · SC6A1_HUMAN)We included GAT-1(A357V) associated with other DEEs as reference (**B**) Pedigree and the genotype. 1069G > A variation resulting in GAT-1(A357T) was found in two siblings, eight-year-old boy and the five-year sister but not in the mother and father. (**C**). Amino acid sequence homology shows that Alanine (A) at residue 357 is highly conserved in GAT-1 in humans (Accession NO.NP_003033.3) and across species as shown in red boxed region. (**D**). Electroencephalogram (EEG) of both the brother and sister carrying the GAT-1(A357T) encoding mutation showing background with slow-waves and high amplitude burst of 3 Hz spike wave discharges and prominent intermediate slowing in drowsiness as well as sleep spindles and bursts of discharges in sleep.

Previous studies have identified mutations in *SLC6A1* associated with a spectrum of epilepsy syndromes and neurodevelopmental disorders^1,2,8,15,16^. We have previously studied *SLC6A1* mutations associated with a wide spectrum of disease phenotypes including MAE, Lennox-Gastaut syndrome, autism, ADHD, neurodevelopmental delay due to various degrees of loss of GAT-1 function and genetic background of affected individuals. To date, most assayed mutations (Fig. 1A) are *de novo*. We have previously reported the impact of GAT-1(V125M) in two half-sisters^17^. Here we identified a 1069 G to A (c.1069G > A) mutation in *SLC6A1* resulting in a change of Alanine 357 to threonine (p.A357T) (Fig. 1B) affecting a boy and a girl with unaffected parents. This variant of unknown significance was not identified in the shared parents, suggesting gonadal mosaicism. The mutant amino acid of Alanine is conserved across species (Fig. 1C), suggesting the variation of the amino acid may have significant impact on the transporter protein conformation and function.

### Clinical features and response to medication in affected siblings

Both the boy and the girl were born without complications nor with any medical or developmental concerns until onset of seizures. The first seizure onset was 1 month of age for the boy and 10 months of age for the girl. Both the brother and sister were diagnosed with generalized epilepsy and exhibited similar EEG features and seizure evolution. They both had slow EEG background with 3–4 Hz high amplitude posterior slowing. Frequent posterior notched slow waves, bursts/discharges with absence seizures were identified in both brother and sister. Both had 3–4 Hz burst discharge during drowsiness (Fig. 1D). Irregular bursts of 2.5–3.5 Hz spike and wave discharges with brief absences with eyelid myoclonia or flutter were reported in both. In addition to seizures, both the brother and sister had global developmental delay and were diagnosed with ASD and ADHD. Challenging behaviors including physical outbursts, screaming and shouting, risky behaviors due to lack of danger awareness, as well as cognitive delay and intellectual disability are reported for both the boy and the girl (Supplementary Table 1).

### AI tools-based protein structural modeling suggests that A357T or A357V mutation in GAT-1 protein reduces the mutant transporter protein stability

**Fig. 2:**
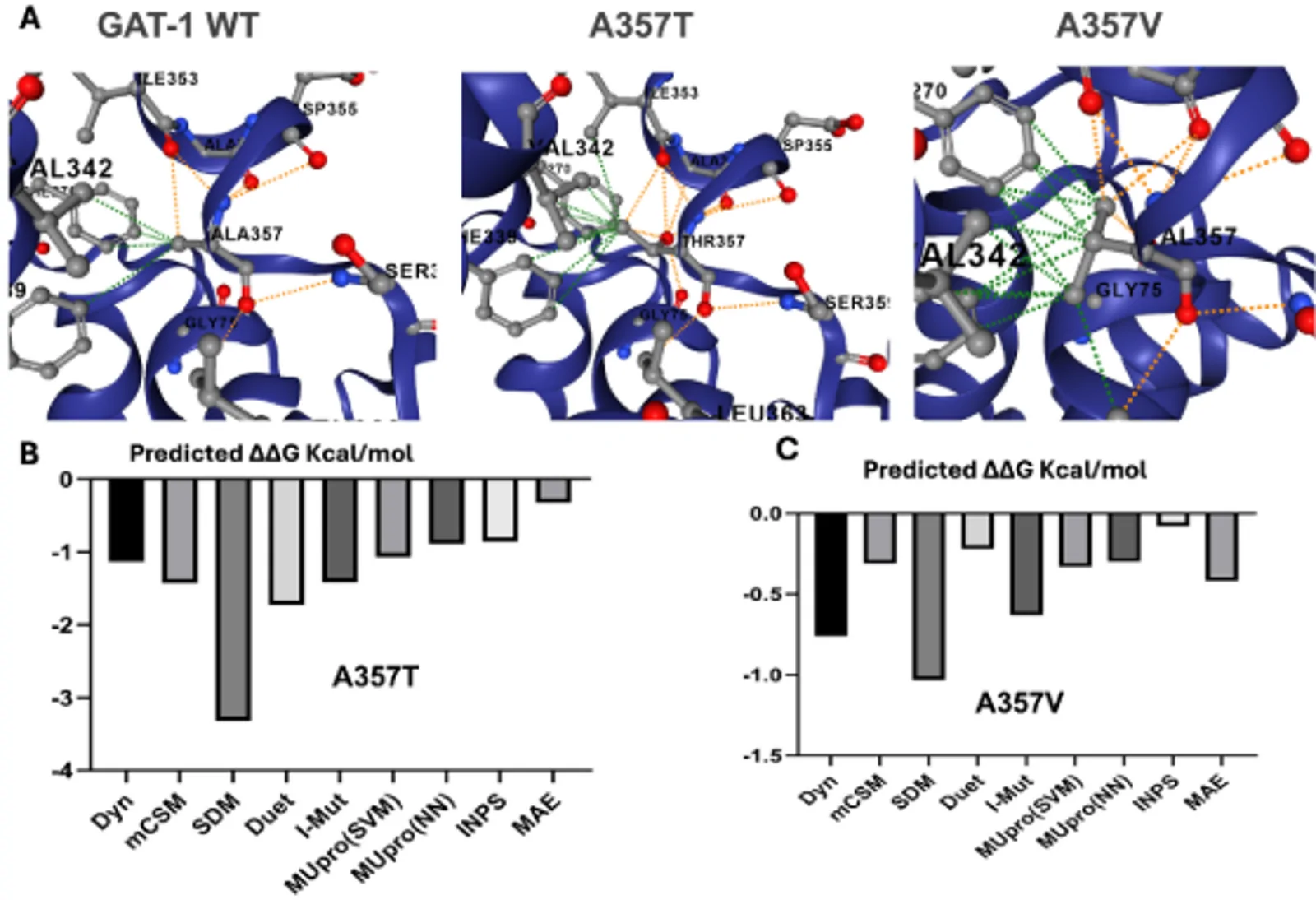
Artificial intelligence tools predict the reduced protein stability of the mutant GAT-1(A357T) and GAT-1(A357V) protein. (**A**). DynaMut2-predicted local environments around residue 357 (yellow arrow) in the wildtype (WT) GAT-1 and the GAT-1(A357T) and GAT-1(A357V) mutants, using the human GAT-1 crystal structure (PDB: 7Y7W). Hydrophobic interactions are shown in green dashed lines; polar interactions (e.g., hydrogen bonds) are shown in orange dashed lines. (B–C) ΔΔG stability predictions for A357T (B) and A357V (C) across multiple computational tools. Negative values indicate destabilization. Methods include DynaMut2 (Dyn), mCSM, SDM, DUET, I-Mutant, MUpro (SVM and NN), INPS, and MAESTROweb (MAE).

We then used AI tools-based protein structural modeling to measure the impact of A357T and A357V mutations in GAT-1 stability. AI-based modeling revealed that both A357T and A357V substitutions in GAT-1 are predicted to reduce thermodynamic stability, with A357T having a stronger effect. As shown in Fig. 2A, DynaMut2 modeling suggested that introducing a threonine at position 357 places a polar hydroxyl group into a predominantly hydrophobic transmembrane pocket, leading to polarity clashes and steric strain. The valine substitution, by contrast, increases hydrophobic bulk, which can enhance local packing but also creates steric hindrance in a confined space.

Across all stability prediction tools (Fig. 2B and C), negative ΔΔG values were observed for both variants, with an average of − 1.4 kcal/mol for A357T and − 0.5 kcal/mol for A357V. While the magnitude of ΔΔG varied between tools, all AI tools predicted destabilization of the mutant protein, with DUET estimating the largest effect for A357T (–3.4 kcal/mol). This consistent pattern across algorithms indicates a robust destabilization signal.

### GAT-1(A357T) and GAT-1(A357V) had impaired GABA reuptake in HEK293T, human iPSC-derived astrocytes, and neurons overexpressing the mutant GAT-1

**Fig. 3:**
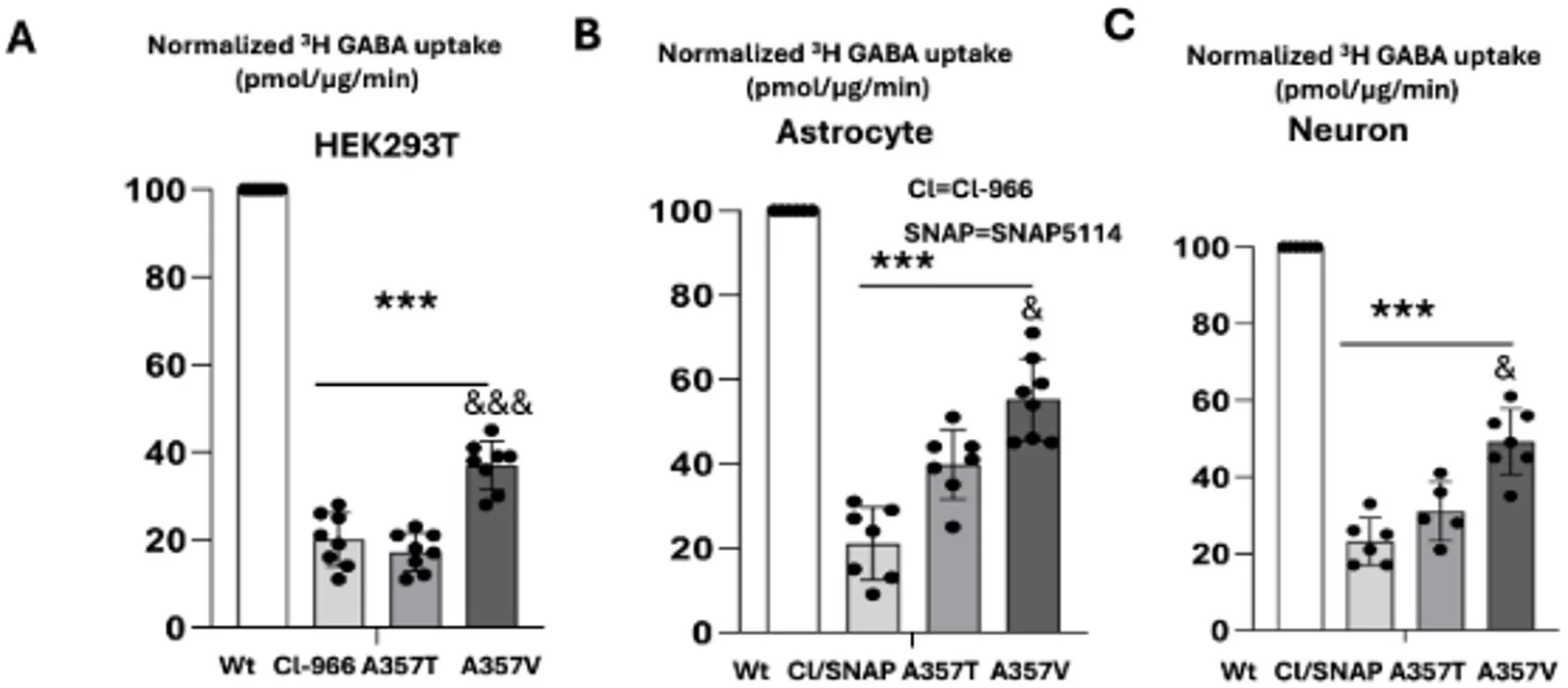
The GABA reuptake function of the mutant GAT-1(A357T) and GAT(A357V) was reduced across cell types. **A.** HEK293T cells under passages 18 were transfected with wildtype GAT-1^YFP^ (wt), or the mutant GAT-1(A357T)^YFP^ cDNAs (1 µg per a 35 mm^2^ dish) for 48 h before ^3^H radioactive GABA uptake assay. **B**,** C**. Human iPSC derived astrocytes (**B**) and cortical neurons (**C**) were transfected with the wildtype, the mutant GAT-1(A357T) or mutant GAT-1(A357V) cDNAs at day 27th after differentiation for astrocytes and 2 months after differentiation for neurons in culture. ^3^H radioactive GABA uptake assay was performed after 2 days of transfection for astrocytes and 5–7 days for neurons. GABA flux was measured after 30 min transport at room temperature. The influx of GABA, expressed in pmol/µg protein/min, was averaged from duplicates of two 35-mm petric dishes for each condition and for each transfection. The average counting was taken as *n* = 1. The untransfected condition was taken as baseline flux, which was subtracted from both the wildtype and the mutant conditions across cell types (**A**). The pmol/µg protein/min in the mutant was then normalized to the wildtype from each experiment, which was arbitrarily set as 100%. (***p < 0.001 vs. wt, & *P* < 0.05; &&& *P* < 0.001; *n* = 7–8 different transfections). The GAT-1 selective inhibitor Cl-966 (100 μm) was applied 30 min across cell types while the GAT-3 inhibitor (SNAP5114 (30µM)) was only applied to astrocytes (Cl/SNAP) during GABA flux. One-way analysis of variance (ANOVA) and Newman-Keuls test.

We then determined the functionality of the wildtype and the mutant GAT-1 transporters in HEK cells, cultured human astrocytes and cortical neurons transfected with either the wildtype or mutant GAT-1 cDNAs. Radioactive ^3^H GABA uptake assay was performed in 48 h after transfection. The measurements in the mutant transporter were then normalized to the cells expressing the wildtype transporter which was taken as 100%. Compared with the wildtype GAT-1^YFP^, both the GAT-1(A357T) and GAT-1(A357V) had reduced ^3^H GABA uptake in HEK293T (wt = 100% vs. 17.25% for A357T and 37% for A357V) (Fig. 3A), human cortical astrocytes (wt = 100% vs. 39.86% for A357T and 55.25% for A357V) (Fig. 3B) and neurons (wt = 100% vs. 31% for A357T and 49.29% for A357V) (Fig. 3C). The GAT-1(A357T) transport activity was lower than the activity of wildtype GAT-1 and was like the wildtype treated with GAT-1 inhibitors Cl-966 (100 µM). However, the GABA uptake in GAT-1(A357V) was higher than the GAT-1(A357T). This is consistent across different cell types, suggesting reduced GABA uptake in both GAT-1(A357T) and GAT-1(A357V) mutant transporters but the GAT-1(A357V) transporter had more remaining GABA uptake function.

### GAT-1(A357T) and GAT-1(A357V) mutant transporters had reduced mutant protein expression and increased immature GAT-1 protein

**Fig. 4:**
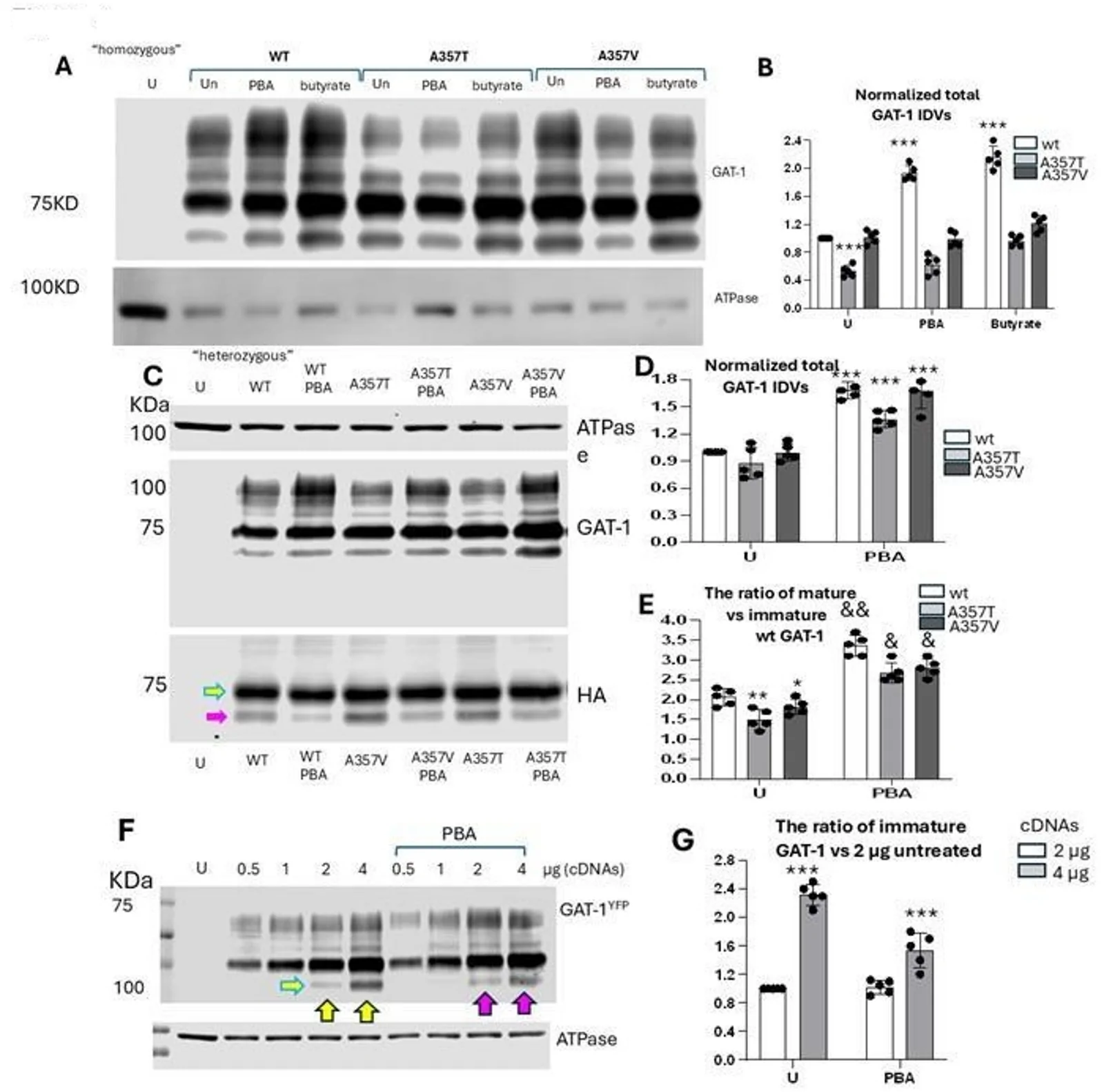
The mature GAT-1 protein was increased but the immature GAT-1 decreased with 4-phenylbutyrate treatment in the mutant transporters. **A**,** B**. HEK293T cells were transfected with wildtype GAT-1^YFP^ (wt), or the mutant GAT-1(A357T)^YFP^ or GAT-1(A357V)^YFP^ cDNAs (3 µg/60 mm^2^) for 48 h. The cells were either treated with vehicle (DMEM 200 µl) or with 4-phenylbutyrate (2 mM) or butyrate (2 mM) for 24 h before harvested for the SDS-PAGE analysis of total lysates (**A**). Membranes were immunoblotted with rabbit anti-GAT-1 (1:300) or anti-HA (1:200). The total protein integrated density values (IDVs) from the total lysates (**B**). **C**,** D**,** E.** HEK293T cells were transfected with GAT-1^HA^ in combination with wildtype GAT-1^YFP^ (wt), or the mutant GAT-1(A357T)^YFP^ or GAT-1(A357V)^YFP^ cDNAs (3 µg/60 mm^2^) for 48 h. The cells were either treated with vehicle (DMEM 200 µl) or with 4-phenylbutyrate (2 mM) for 24 h before harvested for the SDS-PAGE analysis of total lysates for either GAT-1 (**C**, upper panel) or GAT-1^HA^ (**C**, lower panel). The total GAT-1 (**D**) or the ratio of the mature over the immature GAT-1^HA^ protein (**E**) were measured. **F**,** G**. HEK293T cells were transfected with different amounts of GAT-1(A357T)^YFP^ cDNAs. The total amount of cDNAs transfected were normalized with the vector PcDNA. The immature band was indicated by the yellow arrow (**F**). The abundance of the immature GAT-1(A357T)^YFP^ transporter was normalized to immature GAT-1(A357T)^YFP^ from the cells transfected with 2µg of the GAT-1(A357T)^YFP^ cDNAs (**G**). In **B**, the total protein abundance was measured by adding up all the bands between 90–110 KDa. The total protein IDVs of either the wildtype or the mutant was normalized to its loading control and then to the wildtype treated with vehicle. In **C**, the yellow arrow stands for the mature isoform while the pink arrow for the immature isoform of the GAT-1^HA^. In **B**, ***p < 0.001 vs. wt treated with vehicle (U). In **D**, ***p < 0.001 vs. itself untreated. In **E**, * *P* < 0.05; **p < 0.01 vs. wildtype treated with vehicle (wt U); &*P* < 0.05; &&*P* < 0.01 vs. itself untreated. In **D**, ***p < 0.001 vs. itself untreated. In **G**, ***p < 0.001 vs. 2 µg; & *P* < 0.05 4 µg with PBA vs. 4 µg non PBA. *N* = 5 different transfections, Two-way ANOVA with Turkey’s post-hoc test, Values were expressed as mean ± S.E.M).

We then determined the total expression of the GAT-1(A357T) and the GAT-1(A357V). We first expressed the wildtype and the mutant GAT-1 in the mutant condition. The cells were treated with PBA 2 mM or butyrate 2 mM for 24 h. The total lysates of the wildtype and the mutant GAT-1 were analyzed with SDS-PAGE and immunoblotted with GAT-1(A). Compared with the wildtype, which is taken as 1, the GAT-1(A357T) was reduced while there was no difference between the GAT-1(A357V) and the wildtype (Fig. 4A and B). The total amount of GAT-1(A357V) (0.532 ± 0.079) was more than the GAT-1(A357T) (1.018 ± 0.093) (Fig. 4B).

### The wildtype GAT-1 had increased immature protein in both the GAT-1(A357T) and the GAT-1(A357V) but was reduced by PBA

We then expressed the wildtype (WT) GAT-1^HA^ and the mutant GAT-1 in the WT/mutant coexpression condition and determined the mature and immature form of the GAT-1 and the wildtype GAT-1^HA^. As evidenced in our previous study^18^, GAT-1 ran at three bands 108 KDa, 96 KDa and 90 KDa^18^ (Fig. 4C). Although there are multiple bands of GAT-1, PBA increased the 108 KDa (Fig. 4D) in both the mutant (1.36 ± 0.094 for A357T and 1.678 ± 0.197) and the wildtype (1.684 ± 0.094). Consistently, the ratio of the mature over the immature GAT-1 protein was increased in both the wildtype (2.06 ± 0.207 for untreated vs. 3.36 ± 0.261 PBA treated) and the mutant (1.5 ± 0.245 for A357T untreated vs. 2.68 ± 0.249 A357T PBA treated; 1.82 ± 0.192 for A357V untreated vs. 2.8 ± 0.224 A357V PBA treated) (Fig. 4E). We hypothesized that the presence of the immature GAT-1 was due to the deficient folding capacity in the cells. Indeed, in the cells transfected with GAT-1 of different cDNA amounts, only the cells transfected with 2 µg and 4 µg cDNAs had immature GAT-1 (Fig. 4F) while PBA decreased the immature GAT-1 in the cells transfected with 4 µg cDNAs (2.32 ± 0.148 for 4 µg untreated vs. 1.54 ± 0.241 A357T PBA treated) (Fig. 4G).

### GAT-1(A357T) mutant protein had increased endoplasmic reticulum retention while PBA can reduce the ER retained GAT-1 protein

**Fig. 5:**
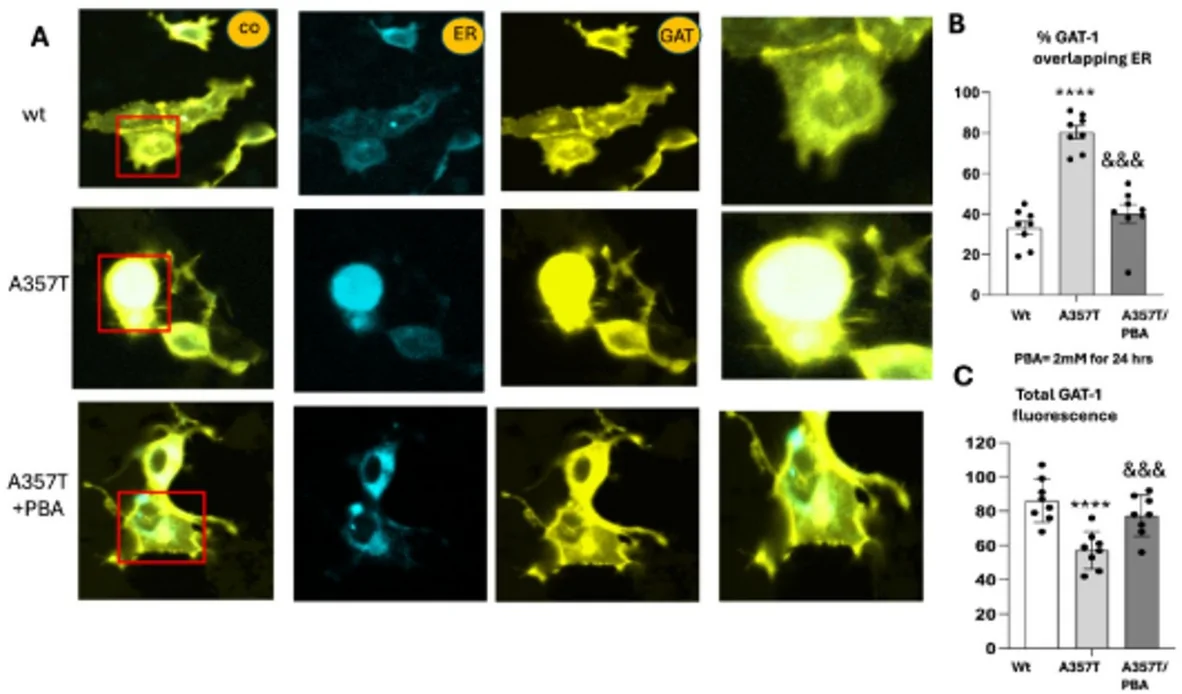
The mutant GAT-1(A357T) c endoplasmic reticulum but were relieved by 4-phenylbutyrate. (**A**)HEK293T cells were transfected with wildtype GAT-1^YFP^ (wt) or the mutant GAT-1(A357T)^YFP^ with the pECFP-ER marker (ER^CFP^) at 1:1 ratio (1 µg:0.5 µg cDNAs) for 48 h. The cells were treated with vehicle or 4-phenylbutyrate (2 mM) for 24 h before confocal microscopy. Live cells were examined under a confocal microscopy with excitation at 458 nm for CFP, 514 nm for YFP. All images were single confocal sections averaged from 8 times to reduce noise, except when otherwise specified. (**B**,** C**) The GAT-1^YFP^ fluorescence overlapping with ER^CFP^ fluorescence (**B**) or the total GAT-1^YFP^ fluorescence (**C**) was quantified by Metamorph with colocalization percentage. (**C**). PBA stands for 4-phenylbutyrate (2 mM) treated for 24 h. (***p < 0.001 A357T vs. wt; &&& *p* < 0.001 A357T + PBA *N* = 8 different transfections. The images from three representative fields per dish were acquired for each transfection, and the mean fluorescence intensity across the three fields was calculated and treated as one biological replicate (*n* = 1). One-way analysis of variance (ANOVA) and Newman-Keuls test was used to determine significance compared to the wt condition and between A357T and A357T/PBA. Values were expressed as mean ± S.E.M).

We have previously identified that increased endoplasmic reticulum (ER) retention of mutant protein due to misfolding and glycosylation arrest is a common phenomenon for mutation across genes affecting GABAergic pathway^1,2,7^. Those ER retention-prone mutant proteins can have either a higher or lower proportion of the total protein level compared with the wildtype. To evaluate the subcellular localization of GAT-1(A357T), we determined the extracellular localization of the mutant GAT-1(A357T) protein by coexpressing GAT-1^YFP^ or GAT-1(A357T)^YFP^ with an ER marker, ER^CFP^ with or without PBA treatment for 24 h. When compared to wildtype, the mutant GAT-1(A357T) had a stronger presence, colocalizing with the ER marker (Fig. 5A). In the cells expressing GAT-1(A357T)^YFP^ with PBA treatment, the GAT-1(A357T)^YFP^ protein expression pattern was comparable to the expression pattern of the wildtype GAT-1 protein. The percent fluorescence signal of GAT-1 overlapping with ER marker ER^CFP^ was higher in the mutant GAT-1(A357T) compared to wildtype (33.13 ± 5.8% for wt vs. 80.38 ± 4.5%) for A357T) (Fig. 5B). The fluorescence signal overlapping with ER marker was lower in the cells expressing GAT-1(A357T) after PBA treatment than GAT-1(A357T) (40% ± 6.7%) without PBA (2mM) treatment (Fig. 5B). We also measured the total GAT-1 fluorescence of the GAT-1 in each condition. We identified that the raw values of total fluorescence intensity of GAT-1(A357T) were lower than the wildtype (86.13 ± 6.4) for wildtype vs. 57.25 ± 8.6 for A357T) while PBA increased the total GAT-1(A357T) fluorescence (77.38 ± 4.5). Increased retention of GAT-1(A357T) within the ER is expected to limit forward trafficking of the transporter to the plasma membrane. Although direct surface labeling was not performed in this study, reduced GAT-1 surface availability is functionally reflected by the marked decrease in GABA uptake observed in cells expressing the A357T variant (Fig. 3). Together, these findings support impaired ER processing and trafficking as the primary mechanism underlying reduced GAT-1 function. PBA rescue is likely via improved GAT-1 trafficking. It is worth noting that cells expressing mutant GAT-1 exhibited a more rounded morphology and pronounced colocalization of GAT-1 with the ER, consistent with our central hypothesis that the mutant GAT-1 is retained within the ER.

### The impaired GABA uptake in the mutant protein was restored by pharmacological or genetic upregulation of chaperones in the ER

**Fig. 6:**
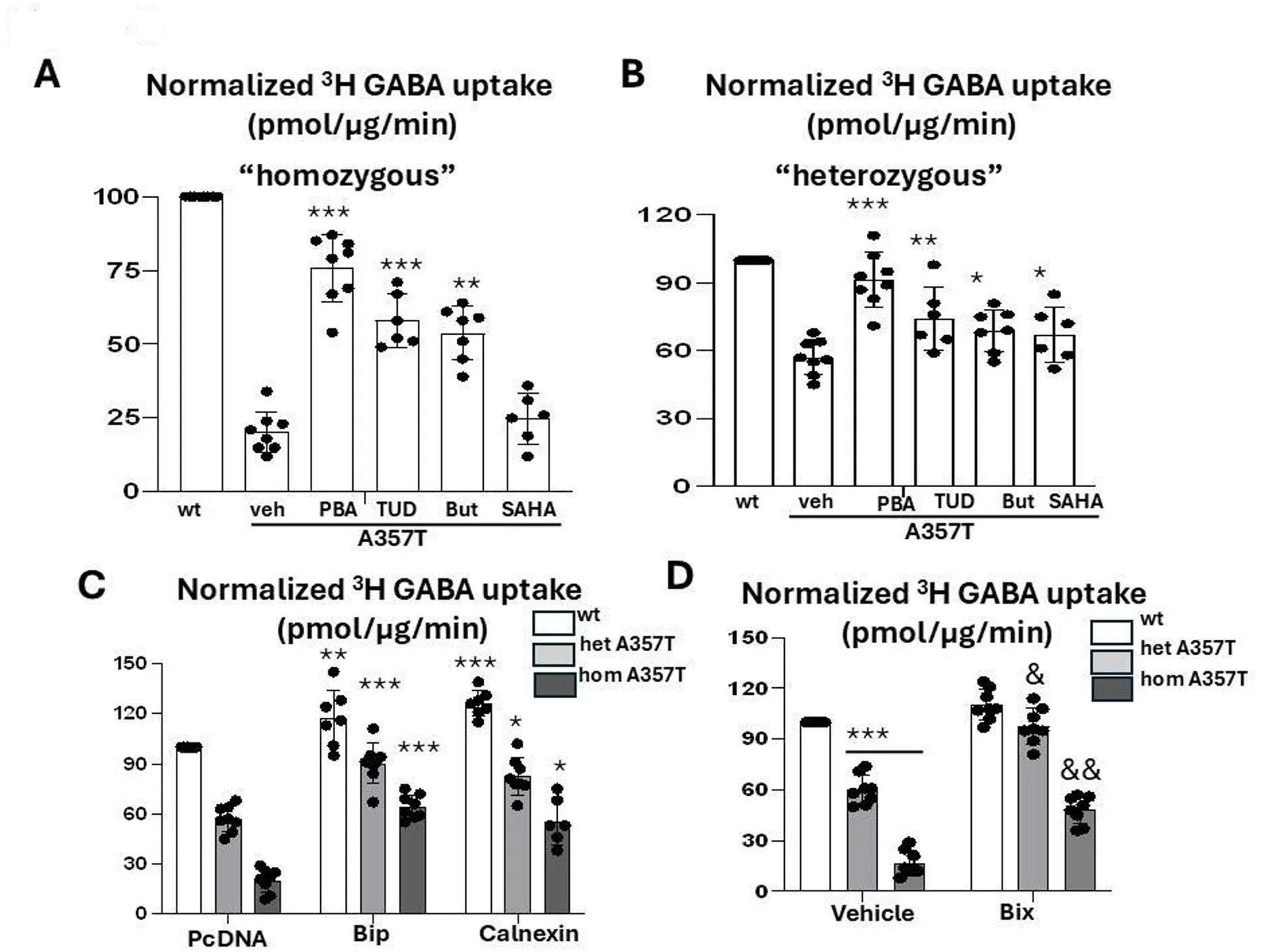
The mutant GAT-1(A357T) mutant transporters were rescued by either pharmacological or genetic upregulation of chaperone proteins. (**A**,** B**) HEK293T cells were transfected with wildtype GAT-1^YFP^ or the mutant GAT-1(A357T)^YFP^ (1 µg cDNAs per dish) (**A**) or with the mixture of the wildtype and the mutant GAT-1 (0.5 µg:0.5 µg cDNAs) (**B**). The cells were then treated with vehicle, 4-phenylbutyrate (2mM), TUDCA (100 µM), butyrate (2mM) and SAHA (2.5 µM) for 24 h before GABA binding experiment. In A, *N* = 7–8 batches of cells; in B, *N* = 6–8 batches of new cells. (**C**). HEK293T cells were cotransfected with the wildtype (Wt), the mixture of the wildtype, the GAT-1(A357T) cDNAs (0.5 µg:0.5 µg) (het) and the mutant GAT-1(A357T) cDNAs (mut) with either PcDNA, Bip or Calnexin at a ratio of 1:1. *N* = 6–8 batches of cells. (**D**). HEK293T cells were cotransfected with the wildtype (Wt), the mixture of the wildtype, the GAT-1(A357T) cDNAs (0.5 µg:0.5 µg) (mixed) and the mutant GAT-1(A357T) cDNAs (mut). The cells were treated with vehicle (DMEM 200 µl) or Bix (10 µM) for 24 h. (In A and B, ***p < 0.001; ** *p* < 0.01 vs. wt; & *P* < 0.05; && *P* < 0.01 vs. A357 (A) or wt/A357T (B). In C, ***p < 0.001; * *p* < 0.05 vs. wt PcDNA ; & *P* < 0.05; && *P* < 0.01 vs. mixed PcDNA; #*P* < 0.05; ###*P* < 0.001 vs. mut PcDNA. In D, ***p < 0.001 vs. wt; &&*P* < 0.01 vs. mixed Veh ; ##*P* < 0.01 vs. mut Veh. *N* = 8 different transfections. Two-way analysis of variance (ANOVA) and Newman-Keuls test was used to determine significance between different conditions. Values were expressed as mean ± S.E.M).

We have previously reported that PBA can restore GABA uptake function in the *SLC6A1* variants and mitigate seizures in the variant bearing mouse models^3^. However, the mechanism of action for PBA is not clear. PBA is known as a chemical chaperone and histone deacetylase (HDAC) inhibitor. However, its precise mechanism of action, whether pharmacochaperoning or HDAC inhibition, remained unclear. Here we compare the effect of PBA with pharmacochaperones such as TUDCA and HDAC inhibitors including both butyrate and SAHA on GABA uptake. We have evaluated whether PBA restores GAT-1(A357T) function through pharmacochaperoning or HDAC inhibition. We first compared the effect on GABA uptake of the mutant GAT-1 under the mutant-only condition in the cells treated with PBA, TUDCA, butyrate and SAHA (Fig. 6A). We then tested the effect of the compounds in the cells expressing the mutant GAT-1 under the WT/mutant coexpression condition (Fig. 6B). In both conditions, PBA and TUDCA increased the GABA uptake while butyrate and SAHA treated cells exhibited inconsistent increase of GABA uptake. This suggests that the effect of PBA on GABA uptake is likely contributed by its pharmacochaperoning effect. We then coexpressed the ER chaperone such as Bip and calnexin with the wildtype or the mutant GAT-1(A357T). Compared with the wildtype (= 100%) or mutant WT/mutant coexpression (mixed) (57.125% ± 7.73%) or mutant (mut) ((20.125% ± 7.1%) GAT-1(A357T) coexpressed with PcDNA, both the wildtype (117.429% ± 16.88% for Bip and 126.143%±7.69% for Calnexin) and the mutant GAT-1(A357T) coexpressed with Bip (90.25% ± 12.245% for mutant and 64.375% ± 7.21% for mut and Calnexin (82.375% ± 11.032)% for mixed and 55.5% ± 13.751%) for mut had increased GABA uptake (Fig. 6C). In the same line, the cells expressing the mutant GAT-1(A357T) treated with Bip activator Bix (10 µM)^6,20^ also had increased GABA uptake in both the wildtype (110 ± 9.19% Bix vs. vehicle) and mutant GAT-1 (60.125% ± 8.69% for het and 16.875% ± 7.29% for mutant treated with vehicle vs. 97.625% ± 10.50% for het and 48.125% ± 8.254% for mutant treated with PBA). Together, this indicates that the upregulation of ER chaperone via either pharmacological or genetic approach can increase the GABA uptake function in the mutant GAT-1(A357T) protein.

## Discussion

### The common and differential phenotypes as well as the antiseizure drug response in affected siblings carrying the same *SLC6A1* mutation

Both the boy and the girl were born healthy until the onset of epilepsy. The first seizure onset for the boy was 1 month, but 10 months old for the girl. Only the boy had hypotonia. Both siblings exhibited similar EEG features and seizure evolution. They both had slow EEG background with 3–4 Hz high amplitude posterior slowing. Frequent posterior notched slow waves, bursts/discharges with absence seizures were identified in both brother and sister. Both had 3–4 Hz burst discharge during drowsiness (Fig. 1D). Irregular bursts of 2.5–3.5 Hz spike and wave discharges with brief absences with eyelid myoclonia were reported in both. In addition to seizures, both the brother and sister were diagnosed with ASD and ADHD, cognitive delay, and intellectual disability. The clinical presentation of the affected children are consistent with previous reports^21–24^ on SLC6A1 variants.

### AI tools predict that reduced protein stability is common across mutant GAT-1 proteins

Because experimental evaluation is often costly and labor-intensive, AI-based tools offer a powerful complementary approach to assess the impact of genetic mutations on protein stability. This transformative capability holds promise for identifying novel therapeutic targets and advancing precision drug discovery strategies in DEEs. We have extensively studied the pathophysiology of GABA_A_ receptors and GAT-1 mutations through molecular assays. In both cell and mouse models, destabilizing mutations can cause a range of cellular and network-level changes, ultimately leading to seizures and abnormal neurobehavioral phenotypes^7^.

In this study, AI-based structural predictions consistently indicated that the A357T and A357V variants in GAT-1 destabilize the transporter, with A357T having a stronger effect. Given that GAT-1 stability is essential for ER exit and surface localization, such destabilizations are consistent with our molecular findings of reduced transporter activity for both variants. Although the computational tools estimate thermodynamic stability rather than directly measuring GABA transport, the strong correlation with experimental results reinforces the link between structural destabilization and functional impairment.

The integration of AI-assisted structural analysis with molecular assays provides a robust framework for evaluating variant pathophysiology. This combined approach enables searching of variants for deeper mechanistic studies and therapeutic screening. While AI-assisted protein stability predictions offer useful structural insight of the mutant protein, they are inherently model-dependent and context-limited, experimental validation remains essential for interpreting the biological consequences of GAT-1 variant proteins. Looking forward, applying this workflow to a broader set of *SLC6A1* variant proteins could reveal shared structural changes that contribute to transporter dysfunction. Such insights may guide exploring modes of actions of pharmacological chaperones, ultimately achieving more targeted and effective treatments for DEEs.

### The mutant GAT-1(A357T) protein is less stable than the GAT-1(A357V) protein

Both AI-assisted tools and experimental approaches indicate that both the GAT-1(A357T) and the GAT-1(A357V) proteins are less stable than the wildtype GAT-1 protein. Importantly, the GAT-1(A357T) protein is more destabilized than the GAT-1(A357V) protein. DynaMut2 suggested that introducing a threonine at position 357 places a polar hydroxyl group into a predominantly hydrophobic transmembrane pocket, leading to polarity clashes and steric strain. The valine substitution, by contrast, increases hydrophobic bulk, which can enhance local packing but also creates steric hindrance in a confined space. Across all stability prediction tools (Fig. 2B and C), negative ΔΔG values were observed for both variants, with an average of − 1.4 kcal/mol for A357T and − 0.5 kcal/mol for A357V. While the magnitude of ΔΔG varied between tools, most methods predicted destabilization, with DUET estimating the largest effect for A357T (–3.4 kcal/mol). This consistent pattern across algorithms indicates a robust destabilization signal of the mutant GAT-1.

### Pharmacochaperoning effect underlies the functional rescue of GABA uptake

Based on the comparison of GABA uptake in the cells treated with PBA, butyrate, TUDCA and SAHA as well as the genetic or pharmacological approaches to directly upregulate ER chaperones (Fig. 6), it is likely that PBA rescues the mutant GAT-1 GABA uptake via pharmacochaperoning effect. This is consistent with our previous findings on a large cohort of SLC6A1 mutations^6^ that pharmacochaperoing is the major driver for GAT-1 functional rescue. Regarding the pathogenicity of SLC6A1 mutations, it is established that most if not all of the *SLC6A1* mutations give rise to mutant GAT-1 with reduced GABA uptake function based on studies in cell and mouse models^1–3,14,17^. Our findings on the GABA uptake of the GAT-1(A357T) and GAT-1(A357V) are consistent with previous studies^1,2^. PBA upregulated the GABA uptake for both the GAT-1(A357T) and GAT-1(A357V) variants. Importantly, we demonstrated that pharmachaperone such as TUDCA can upregulate GABA uptake while the rescue of GABA uptake is more variable in the cells treated with HDAC inhibitors such as butyrate and SAHA.

### The mutant GAT-1 (A357T) had increased GAT-1 protein ER retention that can be relieved by PBA treatment

ER retention and enhanced degradation is a common molecular mechanism for many disease mutations including those in GABA_A_ receptors and GAT-1 associated with variable genetic epilepsy and DEEs. We evaluated the subcellular colocalization of GAT-1(A357T) with coexpression of GAT-1^YFP^ with an ER marker. We compared the expression pattern of the GAT-1(A357T) with or without PBA treatment with the wildtype GAT-1. Our data indicates that the mutant GAT-1(A357T) transporter after PBA treatment was less retained inside ER and the total GAT-1(A357T) was increased, suggesting that more functional GAT-1 was trafficked to the cell membrane.

### PBA reduced the immature GAT-1 in the wildtype when expressed under disease- relevant WT/mutant co-expression condition

Because all the patients are heterozygous, we focused on the heterozygous condition. We identified that the immature form of the wildtype GAT-1 in the WT/mutant co-expression condition was increased. However, PBA treatment reduced the immature GAT-1 of the wildtype allele but increased the ratio of mature vs. immature GAT-1. Because all the patients carry a wildtype allele of GAT-1, the increased mature and reduced immature form of the GAT-1 is likely contributed to the rescue of GABA uptake function and mitigated seizure phenotypes in the mice of *SLC6A1* mutation mediated disorders.

In this work, AI-assisted structural prediction tools were intentionally used as hypothesis-generating aids rather than as a substitute for experimental data. While these computational approaches suggested a destabilizing effect of the A357T variant, definitive conclusions regarding pathogenicity and pharmacochaperone rescue were established through functional uptake assays, protein maturation analyses, and trafficking studies. Together, these findings indicate that computational prediction tools can support variant prioritization but cannot replace experimental validation, which remains indispensable for defining pathogenic mechanisms and guiding precision therapeutic strategies in *SLC6A1*-related epileptic encephalopathies.

In summary, this study has reported for the first time the impact of the same *SLC6A1* variant in siblings of opposite genders and the common and differential clinical features in the siblings. We used both AI tools and experimental approaches to determine mutational impact and the correlation of AI prediction and experimental validation. We identified the molecular defects with a multidisciplinary approach including ^3^H GABA uptake assay and confocal microscopy in cells including astrocytes and neurons. The study provides a critical link for understanding the molecular alterations and clinical phenotypes as well as AI and experimental studies for *SLC6A1* variants. The deficit of GAT-1 function in astrocytes in thalamus could lead to commonly seen EEG features like 3-HZ abnormal discharges in absence seizures and the comorbid attention deficit disorders. Pharmacological treatment with PBA restored GABA uptake in cells expressing mutant GAT-1, consistent with its pharmacochaperoning activity that enhances protein folding, maturation, and forward trafficking, as established in our prior work^2–4^. Together with previous in vitro and in vivo findings^6,25^, these results support pharmacochaperoning as the primary mechanism by which PBA improves GAT-1 function, rather than nonspecific transcriptional or enzymatic effects.

## Supplementary Information

Below is the link to the electronic supplementary material.

Supplementary Material 1

Supplementary Material 2

Supplementary Material 3

## Data Availability

Any raw data of functional assay can be made available upon request.Any clinical information can be made available upon request subject to approval by the appropriate ethical board.

## Abbreviations

GAT-1: GABA transporter 1
ER: Endoplasmic reticulum
MAE: Myoclonic atonic epilepsy
ADHD: Attention deficit hyperactivity disorder
OIRDA: Occipital intermittent rhythmic delta activity

## Experiments and protocols

All methods were carried out in accordance with relevant guidelines and regulations. All experimental protocols were approved by Vanderbilt University biosafety and radiation safety committees.
