# Supplementary material for: Functional characterization and pharmacochaperone rescue of a novel SLC6A1 variant associated with developmental epileptic encephalopathies
Source: Sci Rep. 2026 Oct 7;16:33835. doi: 10.1038/s41598-026-72601-z (PMC13694243; doi:10.1038/s41598-026-72601-z)

Supplementary Figure 2 : Full gel blots of main Figure 4A, 4C, and 4F.

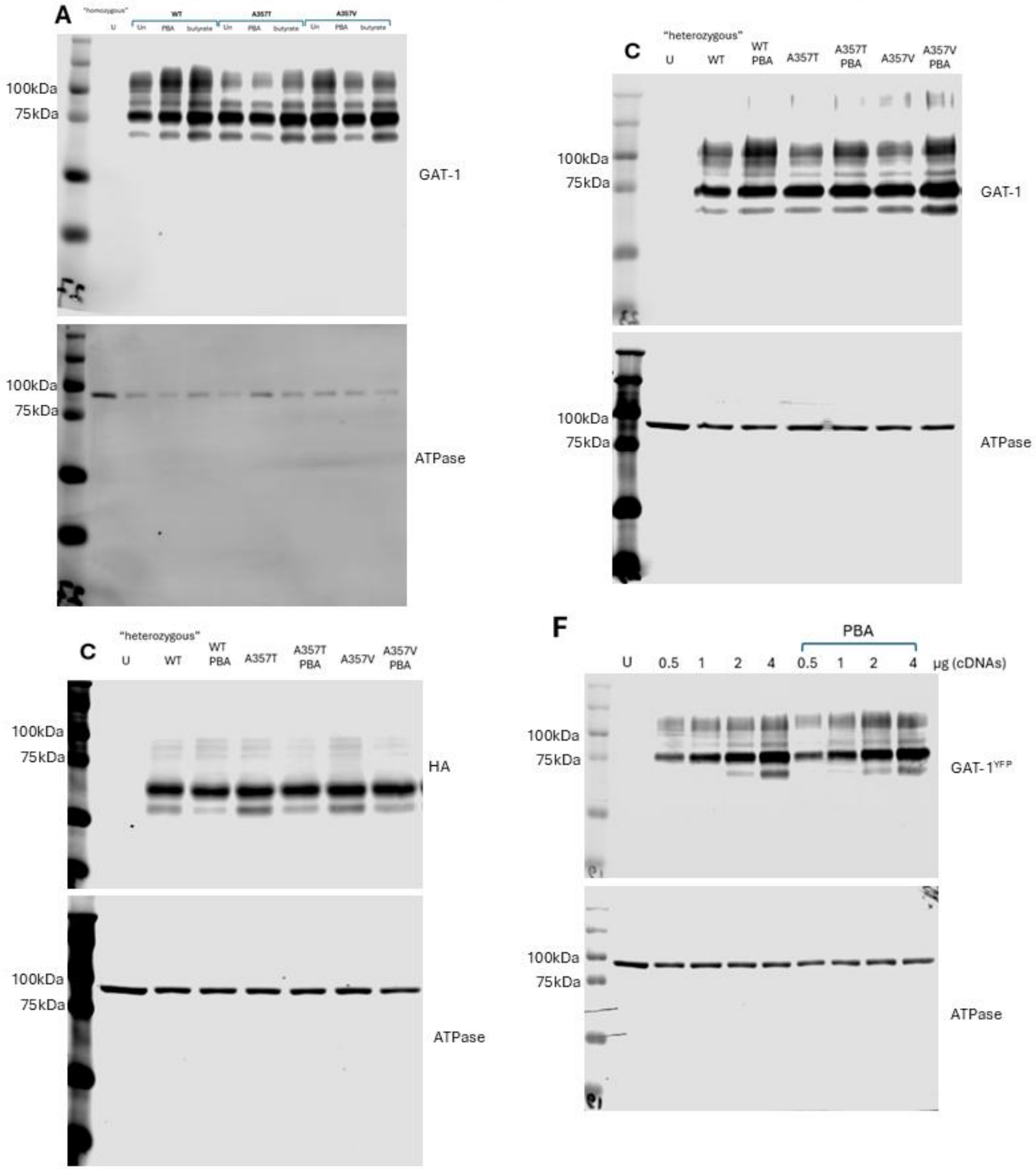

Supplement: Supplementary file 1 [file 41598_2026_72601_MOESM2_ESM.pdf]
