# Supplementary material for: Functional characterization and pharmacochaperone rescue of a novel SLC6A1 variant associated with developmental epileptic encephalopathies
Source: Sci Rep. 2026 Oct 7;16:33835. doi: 10.1038/s41598-026-72601-z (PMC13694243; doi:10.1038/s41598-026-72601-z)
Supplement: Supplementary file 2 [file 41598_2026_72601_MOESM3_ESM.docx]

**Supplementary Table 1**

|  | Patient 1 | Patient 2 |
| --- | --- | --- |
| Age/Sex | 8 year old boy | 5 year old girl |
| Pregnancy | normal | Normal |
| Delivery | 38 weeks assisted by ventouse (no complications) | 39 weeks by ventouse (no complications) |
| Developmental Milestones | 1. Sat up at 6.5 months 2. Crawled at 11 months 3. Independent walking at 22 months- currently some balancing difficulties   Speech delay at 2 years – using occasional individual words | - Crawled at 8 months  - Babbling at 12 months, currently using single words or signs to communicate - First independent steps at 1 year  - currently walking independently with some balance difficulties |
| Past Symptoms | 4. 6 months – episodes of vacant stares with eyes rolling    1. Episodes progressively became longer and occurred multiple times per day    2. By 1 year of age, episodes associated with head drop 5. Between age of 2 and 6 months – developed episodes of exaggerated startle reflex even in absence of obvious trigger | - Eye rolling events starting at 10 months  - Atonic seizures multiple times per day  - Subtle absence seizures |
| EEG Results | 1. **EEG at 21 months** 2. Diffusely slow background with 3-4 Hz posterior slowing 3. Irregular bursts of 2.5-3.5 Hz spike and wave discharges with brief absences with eyelid myoclonia 4. No photosensitivity 5. **EEG at 7 years** 6. Diffuse slowing of background 7. more marked posteriorly with some notched components, especially on eye closure 8. Spike wave discharges enhanced in sleep with irregular generalized bursts with an anterior emphasis 9. No photosensitivity 10. Several eye rolling/opening/eyelid fluttering episodes but not consistently associated with discharges. 11. No eyelid myoclonias 12. **Sleep EEG Results (01 31 2018) (Fig. A-D)** 13. Background shows diffuse slowing, more pronounced posteriorly with notched components on eye closure, consistent with prior recordings (2018 & 2019) 14. Prominent movement and myogenic artifact present 15. Mixed frequencies with dominant 3–4 Hz theta activity and higher amplitudes posteriorly 16. Frequent 2–3 Hz spike/wave bursts, often posterior, sometimes clinical; generalized irregular spike-wave bursts also seen in light sleep with anterior > posterior emphasis (new finding) 17. Several eye rolling/opening/eyelid fluttering episodes noted, not consistently linked to discharges; no eyelid myoclonias, drop attacks, or jerks 18. Seven brief ictal-like 2–3 Hz spike/wave episodes with eye closure/vacant look and hesitation; no drop events or definite seizures captured 19. Hyperventilation and photic stimulation produced no changes; not photosensitive 20. Reported events do not appear ictal 21. EEG pattern differs from sister’s latest study 22. **Sleep EEG (06 30 2019) (Fig. E and F)** 23. **Interictal (Awake):** 24. Prominent 3.5–4.5 Hz high-voltage posterior slowing with eye closure (max ~500µV, R>L), often rhythmic and notched, especially in drowsiness 25. No clinical correlates 26. Passive eye closure during alertness doesn't reproduce prominent slowing, though 5–7 Hz bi-posterior activity (~60µV) is noted 27. Eyes open: symmetric, low-voltage mixed fast activity (5–20 Hz), with some 4–6 Hz bi-posterior slowing less marked than during eye closure   **Drowsy/Sleep:**   28. Posterior slowing attenuates; mixed 4–20 Hz background with fast activity (15–20 Hz) predominating 29. Intermittent bilateral bursts of irregular sharp/spike-slow wave discharges (1 sec), mild frontal bias, no clear lateralisation 30. Occasional 1–2 Hz high-amplitude posterior slow waves (R>L, up to 300µV) 31. Possible vertex sharp waves and moderately symmetrical sleep spindles 32. **Hyperventilation:** Attempted but incomplete; no new features seen **Photic Stimulation:** Occipital response present bilaterally; no other changes   **Ictal:** No events captured  **EEG (03 01 2024) (Fig. G-M)**  **Interictal EEG**   33. Showed background slowing 34. Posterior dominant rhythm was poorly formed 35. Frequent generalized high-amplitude spike-and-wave discharges at 2.5–3.5 Hz 36. These discharges had a maximum over the occipital regions, indicating posterior predominance   **Ictal EEG**   37. During clinical staring spells (likely absence seizures), EEG showed: 38. Generalized spike-and-wave discharges lasting 3–10 seconds 39. Correlated with clinical staring, confirming absence seizure activity   **Sleep EEG**   40. Showed activation of epileptiform discharges 41. More frequent generalized spike-and-wave discharges were observed during sleep 42. This pattern is consistent with generalized epilepsy syndromes and supports diagnosis of a developmental and epileptic encephalopathy 43. Similar results obtained from the EEGs obtained on 11 22 2024 (Fig. N-S) | **EEG at 10 months**   1. Initial EEG: High amplitude posterior slowing 2. Video EEG: Frequent posterior notched slow waves, bursts/discharges with absence seizures   **Repeat EEG at age 4:**   3. Posterior notched slow waves in wakefulness 4. Anterior bursts during sleep 5. Resembles Angelman-like pattern   **Sleep EEG (10 05 20) (Fig. T)**   6. Multiple clusters seven days ago before EEG 7. Aim of EEG – to investigate if there was any epileptogenic activity   **Results**  **Interictal Awake EEG:**   8. Significant movement artefact present. 9. Dominant theta activity at 4 Hz, varying amplitude. 10. Especially high amplitude in posterior regions; irregular. 11. Low-amplitude faster frequencies in background.   **Interictal Drowsy/Sleep EEG:**   12. No clear sleep architecture. 13. Persistent high-amplitude theta activity. 14. Bursts/runs with occasional slightly sharpened contours. 15. Ongoing high-amplitude posterior slow activity. 16. Occasional superimposed beta activity.   **Ictal Findings:**   17. No events recorded during the session.   **Conclusion**   18. EEG affected by artefacts; background shows symmetrical high-amplitude posterior slowing. 19. No clear posterior dominant rhythm or sleep architecture. 20. No epileptiform discharges or clinical events recorded. 21. Subtle eyelid fluttering seen on home video; no events captured on EEG. 22. Findings are nonspecific; recommend clinical correlation, further neurological/genetic workup, and repeat EEG with photic stimulation if symptoms persist.   **24 hr Home Video-telemetry (10 28 20) (Fig. U-V)**   23. Purpose: To investigate if there was any evidence of epilepsy   **Interictal EEG Findings**  **Awake:**   24. Background shows mixed alpha/beta, dominated by high-amplitude theta/delta activity. 25. Bilateral high-amplitude posterior delta (2–4 Hz, 600µV+), irregular or semi-rhythmic. 26. Occasional bursts/runs of delta activity, sometimes lateralized, may have spike/wave features.   **Drowsy/Sleep:**   27. Sleep architecture incomplete but Stage II & IV sleep identifiable; occasional spindles. 28. No clear sharpened waveforms noted. 29. 112 events marked; ~95 brief slow eye-blink episodes (mostly <1 sec), some longer (~5 sec) with eye closure and brief cry.   **During Events:**   30. Eye closure events coincide with bursts of generalized high-amplitude delta activity (up to 5 sec). 31. Posterior notched delta/spike-wave morphology and heart rate increase noted. 32. Similar EEG bursts also seen without clinical events.   **Conclusion & Impression**   33. Background shows symmetrical high-amplitude posterior slowing with variable laterality. 34. Frequent brief episodes of eye blinking, subtle behavioral changes, and rare crying spells were observed. 35. Some events are associated with 3Hz notched posterior delta or brief widespread bursts with spikes; others show no EEG change. 36. EEG findings are unusual, with frequent posterior notched slow waves and intermittent discharges. 37. Some discharges correlate with subtle absence-like events; others occur without clinical signs. 38. Similar EEG and symptoms reported in sibling with *SLC6A1* mutation. 39. Genetic testing and clinical correlation are recommended.   **Sleep EEG Results**  **(02 28 24) (Fig. W-AA)**   40. High-amplitude slow background for age. 41. Frequent posterior notched slow waves with intermixed sharp waves, more prominent on the right, especially with eye closure. 42. Enhanced discharges during sleep, both posterior and anterior, with a mild right-sided bias. 43. Sleep spindles less developed compared to prior study (2020). 44. No seizures, no response to photic stimulation, and not photosensitive. 45. Brief blinking and eye-rolling episodes occurred without EEG correlates; no eyelid myoclonias. 46. Overall atypical EEG with prominent posterior slowing and sleep-related discharges.   **Sleep EEG (11 15 24) (Fig. BB-GG)**   47. Normal symmetrical background activity in both wakefulness and sleep. 48. Frequent notched posterior delta waves (OIRDA) are seen with eye closure. OIRDA are enhanced during drowsiness and sleep, with high-amplitude rhythmic activity and intermixed spikes. 49. - A new finding of anterior sharps (R > L) was noted in wakefulness. 50. In sleep, prolonged runs of high-amplitude, frontally dominant spike-and-slow wave discharges (R > L) are seen, especially in slow-wave sleep. 51. Seven clinical events were captured: four showed typical EEG changes with anterior sharp and slow bursts (2–4 Hz, up to 650 µV, lasting 4–5 s), and three showed no EEG change despite clinical signs like eye blinking or rolling. 52. No seizures or eyelid myoclonias were recorded during photic stimulation. 53. No photosensitivity noted. 54. Hyperventilation was not completed. 55. Findings are consistent with generalized epilepsy with absences, similar to her brother. |
| Other Test Results | MRI at 2 years   1. Normal   Cognitive Assessment with Wechsler Intelligence Scale for Children 5^th^ Edition   1. Full scale IQ - 51 (0.1st Percentile, Extremely Low Range) 2. Verbal Comprehension Index - 62 (1st Percentile; Extremely Low Range) 3. Visual Spatial Index - 57 (0.2nd Percentile, Extremely Low Range) 4. Fluid Reasoning - 64 (1st Percentile; Extremely Low Range) 5. Working Memory Index - 62 (1st Percentile; Extremely Low Range) 6. Processing Speed Index - 53 (0.1st Percentile; Extremely Low Range) 7. Evaluation of speech by Clinical Evaluation of Language Fundamentals –CELF 5 uk ( 5-8) has shown Receptive Language scores at 73 and Language Content at 65 (both below average). 8. Overall functional skills on the ABAS-3 - “Very Low” range (General Adaptive Composite of 70; 2nd centile) 9. |  |
| Past Treatments | 10. Sodium valproate followed by Ethosuximide     1. Unsuccessful 11. Levetiracetam initiated without effect, subsequently Clobazam     1. Contributed to seizure improvement     2. Seizures had been exacerbated by fatigue and before antiseizure medication doses 12. Lamotrigine (switched from Levetiracetam) at 3 years of age     1. Resulted in reduction of intensity and frequency of – seizures 13. Ketogenic diet considered but not initiated due to expected difficulties with compliance | - Lamotrigine (stopped due to rash)  - Levetiracetam (partial improvement)  - Topiramate (minor improvement of atonic seizures) |
| Current Symptoms | 14. Up to 35 episodes of seizures daily | - Continued absence seizures  - PICA behavior  - Behavioral difficulties  - Diagnosed with ASD and ADHD |
| Current Treatments | 15. Lamotrigine 7.6mg/kg/day 16. Clobazam (0.5mg/kg/day) 17. methylphenidate 18mg OD 18. Risperidone 0.25mg OD | - Levetiracetam 33 mg/kg/day  - Clobazam 0.3 mg/kg/day  - Topiramate 2 mg/kg/day   19. Methylphenidate 10mg OD |
| Diagnosis | 20. *SLC6A1*-related neurodevelopmental disorder with: 21. Epilepsy with atypical absences and previously eyelid myoclonia 22. Attention Deficit Hyperactivity disorder (ADHD) 23. Autistic Spectrum Disorder (ASM)   moderate intellectual (learning)disability | - *SLC6A1*-related neurodevelopmental disorder with   24. Generalised Epilepsy with absences  - Autism Spectrum Disorder (ASD) - Attention Deficit Hyperactivity Disorder (ADHD) 25. moderate intellectual (learning)disability - Developmental delay - PICA |

**Supplementary Table 1. Clinical features of the patients with *SLC6A1* (A357T) variant.**

**Supplementary Video 1. Video of 360° rotation animation of GAT-1 protein model with A357T or A357V (pink) mutation.**
